# Crystalloid Coload Reduced the Incidence of Hypotension in Spinal Anesthesia for Cesarean Delivery, When Compared to Crystalloid Preload: A Meta-Analysis

**DOI:** 10.1155/2017/3462529

**Published:** 2017-12-17

**Authors:** Hai-Fang Ni, Hua-yue Liu, Juan Zhang, Ke Peng, Fu-Hai Ji

**Affiliations:** Department of Anesthesiology, First Affiliated Hospital of Soochow University, No. 188 Shizi Street, Suzhou, Jiangsu 215006, China

## Abstract

**Objective:**

To determine whether crystalloid infusion just after intrathecal injection (coload) would be better than infusion before anesthesia (preload) for hypotension prophylaxis in spinal anesthesia for cesarean delivery.

**Methods:**

We searched PubMed, EMBASE, Cochrane Central Register of Controlled Trials, and other databases for randomized controlled trials comparing coload of crystalloid with preload in parturients receiving spinal anesthesia for cesarean delivery. Primary outcome was intraoperative incidence of hypotension. Other outcomes were intraoperative need for vasopressors, hemodynamic variables, neonatal outcomes (umbilical artery pH and Apgar scores), and the incidence of maternal nausea and vomiting. We used RevMan 5.2 and STATA 12.0 for the data analyses.

**Results:**

Ten studies with 824 cases were included. The incidence of hypotension was significantly higher in the preload group compared with the coload group (57.8% versus 47.1%, odds ratio [OR] = 1.62, 95% confidence interval [CI] = 1.11–2.37, and *P* = 0.01). More patients needed intraoperative vasopressors (OR = 1.71, 95% CI = 1.07–2.04, and *P* = 0.02) when receiving crystalloid preload. In addition, the incidence of nausea and vomiting was higher in the preload group (OR = 3.40, 95% CI = 1.88–6.16, and *P* < 0.0001). There were no differences in neonatal outcomes between the groups.

**Conclusions:**

For parturients receiving crystalloid loading in spinal anesthesia for cesarean delivery, coload strategy is superior to preload for the prevention of maternal hypotension.

## 1. Introduction

Cesarean section is one of the most commonly performed surgical procedures worldwide, and 80–90% of them are performed under spinal anesthesia [1]. During the procedures, maternal hypotension is a major complication with the incidence up to 60–70% [2, 3]. The risk factors for hypotension are preoperative hypertension, age, type of anesthesia, and the infant weight [4]. Besides, pregnant women are characterized by increased sympathetic versus parasympathetic activities [5], contributing to the sensitivity to spinal block and vasodilatation [6].

Prolonged hypotension leads to organ ischemia, uteroplacental hypoperfusion loss of consciousness, and cardiovascular collapse [7]. Fluid administration is a daily practice to prevent and treat maternal hypotension. However, the optimal fluid and timing of infusion are yet to be determined. Some studies showed that colloids may be more effective than crystalloids for preventing hypotension [8, 9]. As for colloids, the preload group had lower incidence of hypotension than the coload group [10], but the administration of additional 0.5 L offered no added benefits [6]. However, there are several disadvantages associated with colloids, such as cost, allergic reactions, and their effects on coagulation. As a result, crystalloids are still preferred by many anesthesiologists.

The timing of crystalloid infusion is of great importance because it distributes rapidly into the extracellular space and the volume expanding effect is maximal at the early stage. Traditionally, preload of fluids is used to prevent hypotension in spinal anesthesia, but the efficacy has been questioned. Studies found that fluid coload at the time of actual block during spinal anesthesia was more effective [11, 12]. A previous meta-analysis suggested that the timing of fluid loading did not influence the incidence of hypotension [13], but it combined crystalloid and colloid with only a limited data for crystalloid. In this meta-analysis, we therefore compared coload of crystalloid with preload to determine the optimal timing of infusion for preventing hypotension in spinal anesthesia for cesarean section.

## 2. Material and Methods

### 2.1. Search Strategy

We adhered to the guidelines of the Cochrane Handbook for Systematic Reviews of Interventions throughout this meta-analysis. We searched all relevant trials in the following databases: PubMed, EMBASE, Cochrane Central Register of Controlled Trials, Research Gate, and LILACS, without language or publication date restrictions. The search strategy for PubMed and EMBASE is shown in Supplementary Table 1. Additional studies were retrieved by review of the reference lists from relevant articles.

### 2.2. Inclusion Criteria

- Randomized controlled trial (RCT)
- Healthy parturients scheduled for cesarean delivery under spinal anesthesia
- Use of crystalloids for preload compared to coload
- Outcome measures including intraoperative hypotension, need for vasopressors, intraoperative hemodynamic variables such as heart rate (HR), systolic blood pressure (SBP), and mean arterial pressure (MAP), neonatal outcomes (pH of umbilical artery and Apgar scores), and the incidence of maternal nausea and vomiting

### 2.3. Primary and Secondary Outcomes

The primary outcome was the incidence of hypotension. Secondary outcome measures were need for vasopressors, intraoperative HR, SBP, and MAP, umbilical artery pH, Apgar scores, and nausea and vomiting.

### 2.4. Data Extraction

Data were extracted independently by two investigators and any discrepancy was resolved by group consensus. The following data were extracted: author, publication year, sample size, study design (randomization, blind, allocation concealment, and follow-up), anesthesia, interventions, and outcome measures of interest. The authors of the included studies were contacted for additional information if data was not available from the text.

### 2.5. Study Quality Assessment

The risk of bias was evaluated by two authors independently with the Cochrane Collaboration tool [22]. For each domain, the risk of bias was judged as “high,” “low,” or “unclear.” A trial was considered to have a high risk of bias when one or more domains were at high risk and a low risk of bias when all domains were at low risk. Otherwise, it was judged to have an unclear risk of bias. Any discrepancy over bias assessment was resolved by group discussion.

### 2.6. Statistical Methods

We performed analyses using the RevMan 5.2 (the Cochrane Collaboration, Copenhagen, Denmark) and STATA 12.0 (Stata Corp, College Station, TX). For continuous data, mean difference (MD) with 95% confidence intervals (CIs) was used; for dichotomous outcomes, odds ratio (OR) with 95% CIs was used. We evaluated the statistical heterogeneity of the results with the chi-squared test and the *I* ^2^ statistic, with *I* ^2^ > 50% indicating significant heterogeneity [23]. A random-effects model was used in this meta-analysis [24]. Publication bias was evaluated using a funnel plot. Sensitivity analysis was performed to assess the effect of a single comparison on the overall estimates. A *P* value of <0.05 was considered to be statistically significant.

When range or interquartile range was reported, we estimated the standard deviation as range/4 (range = maximum value–minimum value) or interquartile range/1.35 (interquartile range = Q3–Q1, with Q1 and Q3 representing the first and third quartiles, resp.) [25]. When standard error or CI was reported, standard deviation was calculated with the calculator of RevMan. To increase the robustness of results, data were pooled when at least 3 trials were included for an outcome.

## 3. Results

### 3.1. Study Selection and Characteristics

**Figure 1:**
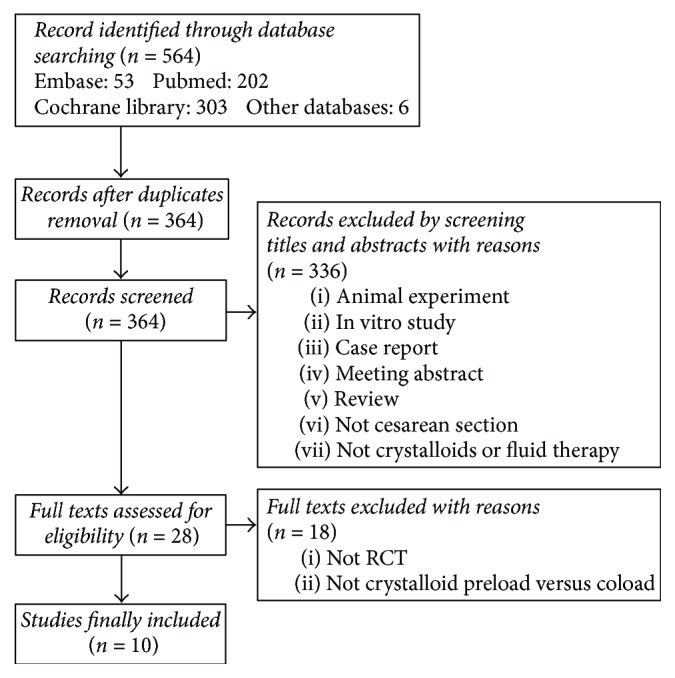
Flow diagram of study selection.

The flow diagram is shown in Figure 1. Ten trials [11, 12, 14–21] with 824 patients were eligible for inclusion into this study. The characteristics are summarized in Table 1. These studies were published from 2004 to 2017 with population sizes ranging from 50 to 120. All studies applied spinal anesthesia for cesarean section. Nine studies enrolled healthy parturients scheduled for elective surgery and one for emergency delivery.

In these studies, hypotension was defined as a 20% decrease from baseline in MAP or SBP, or SBP < 90 mmHg. Seven studies [11, 12, 15–18, 21] used ephedrine to treat intraoperative hypotension, two studies [14, 19] used ephedrine or phenylephrine, and mephentermine was selected in one study [20]. Seven studies [11, 12, 14, 16, 17, 19, 20] used vasopressors when patients developed hypotension, while one study [18] combined crystalloid boluses and ephedrine. Nine studies [11, 12, 14–18, 20, 21] recorded the number of patients with hypotension throughout the surgery, and one study [19] recorded hypotension at 3 and 5 min after anesthesia induction.

### 3.2. Incidence of Hypotension and Need for Vasopressors

**Figure 2:**
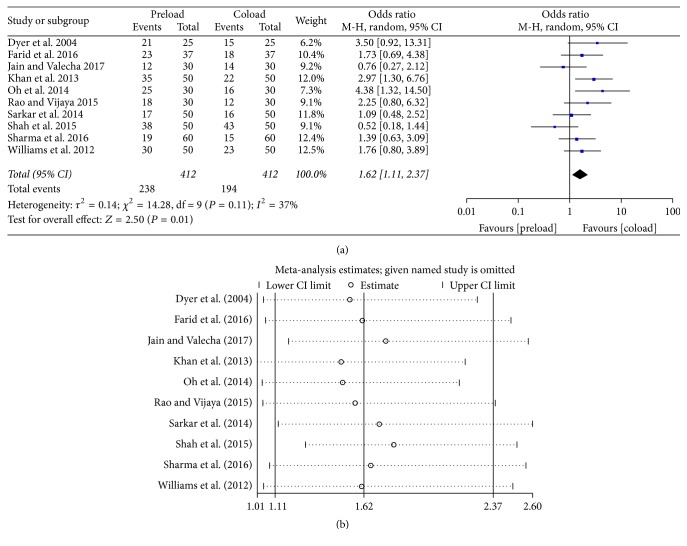
Intraoperative hypotension: (a) forest plot; (b) sensitivity analysis.

**Figure 3:**
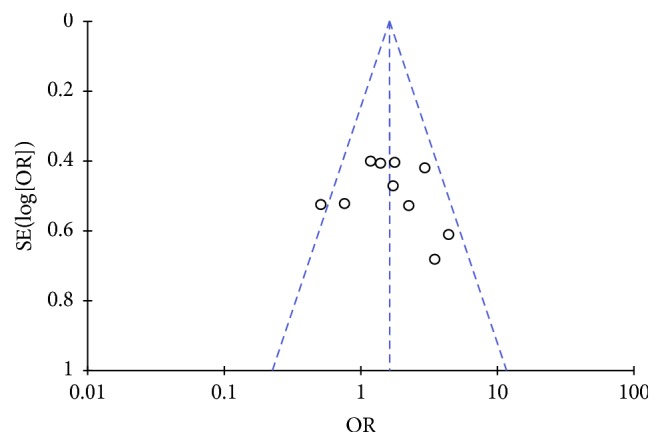
Funnel plot with hypotension as an endpoint.

Pooled data from ten studies [11, 12, 14–21] showed that patients in the crystalloid preload group had more hypotensive episodes than those in the coload group (57.8% versus 47.1%, OR = 1.62, 95% CI = 1.11–2.37, and *P* = 0.01) (Figure 2(a)). Sensitivity analysis reflected that these findings were robust (Figure 2(b)), with pooled ORs ranging from 1.49 (95% CI = 1.01–1.29) to 1.80 (95% CI = 2.18–2.60). The funnel plot with hypotension as an endpoint appeared symmetrical, suggesting that publication bias might not affect the results (Figure 3).

**Figure 4:**
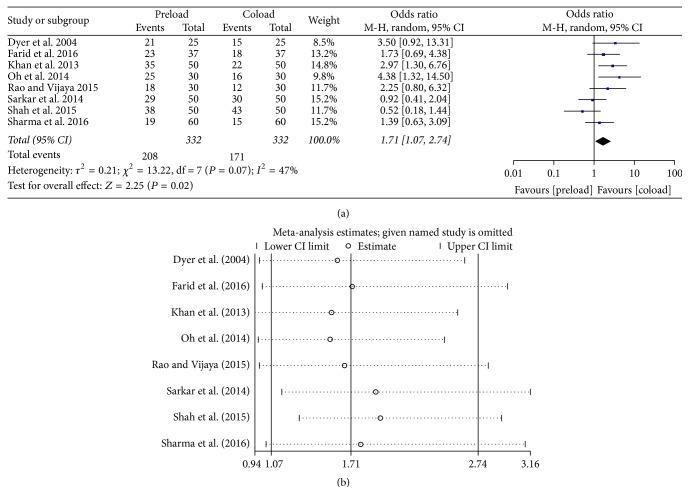
Intraoperative need for vasopressors: (a) forest plot; (b) sensitivity analysis.

Eight studies [11, 12, 14, 16–20] compared the needs for vasopressors between the preload and coload groups. The results indicated a significant increase in the need for vasopressors when patients received fluid preload (OR = 1.71, 95% CI = 1.07–2.04, and *P* = 0.02) (Figure 4(a)). Sensitivity analysis reflected that these findings were robust (Figure 4(b)), with pooled ORs ranging from 1.54 (95% CI = 0.94–1.30) to 1.95 (95% CI = 2.47–3.16).

### 3.3. Hemodynamic Variables

**Figure 5:**
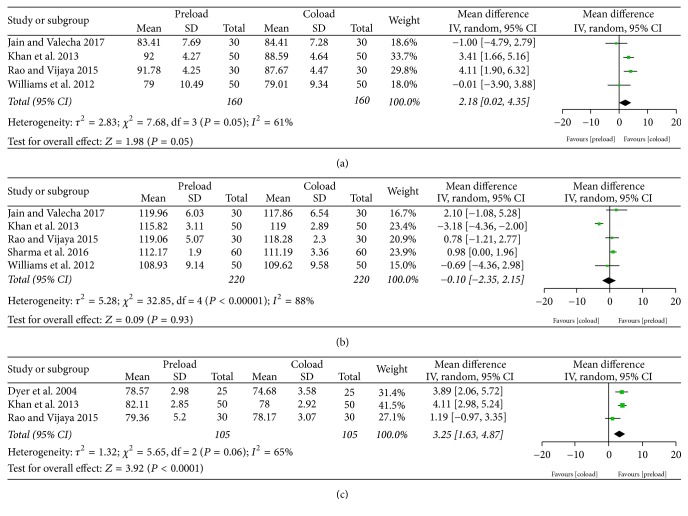
Hemodynamic variables: (a) heart rate and (b) systolic blood pressure during 60 min after spinal anesthesia; (c) mean arterial pressure during 20 min after spinal anesthesia.

Intraoperative HR, SBP, and MAP are shown in Figure 5. Four studies [12, 15, 16, 21] reporting on HR during 60 min after spinal anesthesia showed a higher HR in the preload group (MD = 2.18 beats/min, 95% CI = 0.02–4.35, and *P* = 0.05). Five studies [12, 15, 16, 20, 21] on SBP found no significant difference between the groups. Additionally, the preload group had higher MAP during 20 min after spinal anesthesia (MD = 3.25 mmHg, 95% CI = 1.63–4.87, and *P* < 0.0001) [11, 12, 16].

### 3.4. Other Outcomes

**Figure 6:**
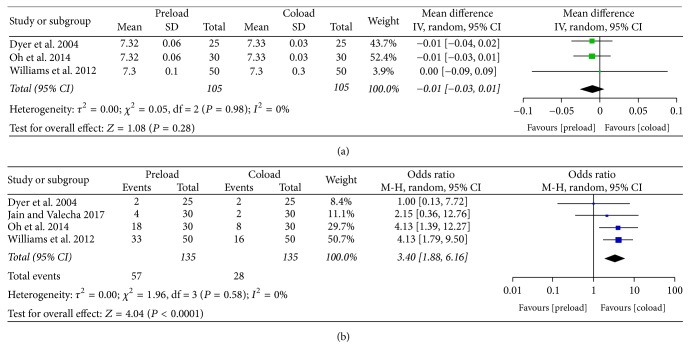
Other outcomes: (a) umbilical arterial pH; (b) nausea and vomiting.

There was no significant difference in umbilical arterial pH between the two groups (Figure 6(a)). Seven studies [11, 12, 15–18, 21] analyzed Apgar scores, and none of them reported Apgar scores < 7 at 5 min. Data from 4 studies [11, 15, 17, 21] showed that the incidence of nausea and vomiting was higher in the preload group (OR = 3.40, 95% CI = 1.88–6.16, and *P* < 0.0001) (Figure 6(b)).

### 3.5. Risk of Bias Assessment

The risk of bias assessment is presented in Table 2. Overall, all studies were double-blinded and randomized. Two studies adequately reported the random sequence generation [15, 19], and five trials clearly reported the allocation concealment [11, 15, 17, 20, 21].

## 4. Discussion

The results of this meta-analysis suggested that coload infusion of crystalloid reduced the incidence of hypotension compared to preload in parturients receiving spinal anesthesia for cesarean delivery. The superiority of coload was further evidenced by a decreased need for vasopressors and a lower incidence of nausea and vomiting.

Crystalloid preload is at times ineffective for preventing hypotension. A previous study by Rout et al. reported that crystalloid preload led to a significant increase in central venous pressure after spinal anesthesia for cesarean section, but the incidence of hypotension was not reduced [26]. The study by Mercier compared four methods of intravascular fluid loading by combining different types of fluid (crystalloid versus colloid) and the timing of administration (preload versus coload). They found that crystalloid preloading or no fluid administration was less likely effective than crystalloid coload for preventing hypotension [27]. According to Starling's law, the exchange of fluid is determined by the capillary and interstitial fluid hydraulic pressure and oncotic pressure [28]. The capillary hydraulic pressure increases over time during crystalloid infusion, which may lead to increased hydraulic pressure difference and fluid filtration from plasma into interstitium. An animal experiment on normovolemic sheep found that the maximum intravascular volume expansion was 27% after infusion, and 15% after 10 min and 7% after 30 min, which indicated a rapid redistribution of crystalloid [29]. Compared with crystalloid preload, coload could help reduce intraoperative hypotension mainly due to the delayed infusion time.

Besides, Pouta et al. suggested that crystalloid preload may induce atrial natriuretic peptide secretion, resulting in peripheral vasodilatation followed by an increased rate of excretion of fluid [30]. Natriuretic peptide type C is a potent vasodilator produced in the endothelium of great vessels [31]. Further fluid loading does not increase the intravascular volume at the time of maximum vasodilation [32]. Atrial natriuretic peptide may even lower blood pressure because of its natriuretic, diuretic, and vasodilatory effects [33]. On the other hand, Ewaldsson and Hahn's study on volume kinetics of Ringer's solution showed that the arterial pressure was better maintained by a fluid bolus just after anesthesia induction compared to preload [34].

In this study, we found that the value of mean HR was lower in the coload group during 60 min after spinal anesthesia, with a lower value of MAP. This inconsistency may be due to various definitions of hypotension, local anesthetics used, types of vasopressors, and infusion rate of crystalloids. During hemodynamic changes, nausea and vomiting often occur. This meta-analysis also showed that the incidence of nausea and vomiting was lower in the coload group. In the previous meta-analysis by Banerjee et al., there was no difference in the nausea and vomiting between preload and coload regimens [13].

Regarding the neonatal outcomes, umbilical arterial pH is sensitive to detecting fetal hypoxia, which indicates the hemostasis at birth. In this study, we did not detect any significant difference in umbilical arterial pH between the groups, and none of the included studies reported Apgar scores < 7 at 5 min. However, the number of cases included for the outcomes is small. Thus, more studies are needed to ascertain the effects of crystalloid loading on neonatal outcomes.

This study has several limitations. First, all included studies have a relatively small sample size. Second, heterogeneity was detected in the outcomes of intraoperative hemodynamic variables, indicating the differences in the definitions of hypotension and the use of vasopressors; therefore, these results need to be interpreted with caution. However, we performed the sensitivity analyses and found the current results were unlikely affected by one single study. Last, this study failed to detect any beneficial effects of crystalloid infusion regimens on long-term outcomes after cesarean delivery. Further studies with larger sample size investigating the short-term as well as long-term outcomes in this population are required.

## 5. Conclusion

For parturients receiving crystalloid loading in spinal anesthesia for cesarean delivery, coload strategy reduced the incidence of intraoperative maternal hypotension and the need for vasopressors.

## Figures and Tables

**Table 1:** Study characteristics.

Study	Group (number of patients)	Anesthesia	Procedure	Study protocol	Vasopressor	Outcome measures
Dyer et al. 2004 [11]	(1) Preload (25) (2) Coload (25)	Spinal anesthesia	Elective cesarean section	(1) 20 ml/kg modified Ringer's lactate over 20 min before anesthesia (2) 20 ml/kg modified Ringer's lactate by rapid infusion at time of CSF identification	Ephedrine (5 mg)	Hypotension, MAP, need for vasopressors, umbilical arterial pH, Apgar score, nausea and vomiting

Farid et al. 2016 [14]	(1) Preload (37) (2) Coload (37)	Spinal anesthesia	Elective cesarean section	(1) 15 ml/kg Ringer's lactate over 20 min before anesthesia (2) 15 ml/kg Ringer's lactate at time of anesthesia administration	Ephedrine or phenylephrine (dose not provided)	Hypotension, need for vasopressors

Jain and Valecha 2017 [15]	(1) Preload (30) (2) Coload (30)	Spinal anesthesia	Elective cesarean section	(1) 15 ml/kg Ringer's lactate over 20 min before anesthesia (2) 15 ml/kg Ringer's lactate over 20 min at time of CSF identification	Ephedrine (3 mg)	Hypotension, HR, SBP, Apgar score

Khan et al. 2013 [16]	(1) Preload (50) (2) Coload (50)	Spinal anesthesia	Elective cesarean section	(1) 20 ml/kg Ringer's lactate over 20 min before anesthesia (2) 20 ml/kg Ringer's lactate by rapid infusion at time of CSF identification	Ephedrine (5 mg)	Hypotension, HR, SBP, MAP, need for vasopressors, Apgar score

Oh et al. 2014 [17]	(1) Preload (30) (2) Coload (30)	Spinal anesthesia	Elective cesarean section	(1) 15 ml/kg Ringer's lactate by rapid infusion before anesthesia (2) 15 ml/kg Ringer's lactate after intrathecal injection	Ephedrine (5 mg)	Hypotension, need for vasopressors, umbilical arterial pH, Apgar score, nausea and vomiting

Rao and Vijaya 2015 [12]	(1) Preload (30) (2) Coload (30)	Spinal anesthesia	Elective cesarean section	(1) 15 ml/kg Ringer's lactate over 20 min before anesthesia (2) 15 ml/kg Ringer's lactate by rapid infusion at time of CSF identification	Ephedrine (6 mg)	Hypotension, HR, SBP, MAP, need for vasopressors, Apgar score

Sarkar et al. 2014 [18]	(1) Preload (50) (2) Coload (50)	Spinal anesthesia	Emergency cesarean section	(1) 15 ml/kg Ringer's lactate over 20 min before anesthesia (2) 15 ml/kg Ringer's lactate over 20 min after anesthesia	Ephedrine (3 mg)	Hypotension, need for vasopressors, Apgar score

Shah et al. 2015 [19]	(1) Preload (50) (2) Coload (50)	Spinal anesthesia	Elective cesarean section	(1) 10 ml/kg Ringer's lactate over 15 min before anesthesia (2) 10 ml/kg Ringer's lactate at time of CSF identification	Ephedrine or phenylephrine (dose not provided)	Hypotension, need for vasopressors

Sharma et al. 2016 [20]	(1) Preload (60) (2) Coload (60)	Spinal anesthesia	Elective cesarean section	(1) 20 ml/kg Ringer's lactate over 20 min before anesthesia (2) 20 ml/kg Ringer's lactate by rapid infusion after intrathecal injection	Mephentermine (3 mg)	Hypotension, need for vasopressors, SBP

Williams et al. 2012 [21]	(1) Preload (50) (2) Coload (50)	Spinal anesthesia	Elective cesarean section	(1) 15 ml/kg Ringer's lactate over 20 min before anesthesia (2) 15 ml/kg Ringer's lactate over 20 min at time of CSF identification	Ephedrine (3 mg)	Hypotension, HR, SBP, umbilical arterial pH, Apgar score, nausea and vomiting

CSF, cerebrospinal fluid; HR, heart rate; SBP, systolic blood pressure; MAP, mean arterial pressure.

**Table 2:** Risk of bias.

Studies	Random sequence generation (selection bias)	Allocation concealment (selection bias)	Blinding of participants and personnel (performance bias)	Blinding of outcome assessment (detection bias)	Incomplete outcome data (attrition bias)	Selective reporting (reporting bias)
Dyer et al. 2004 [11]	Unclear	Low	Low	Low	Low	Unclear
Farid et al. 2016 [14]	Unclear	Unclear	Low	Low	Low	Low
Jain and Valecha 2017 [15]	Low	Low	Low	Low	Low	Low
Khan et al. 2013 [16]	Unclear	Unclear	Low	Low	Low	Unclear
Oh et al. 2014 [17]	Unclear	Low	High	Low	Low	Low
Rao and Vijaya 2015 [12]	Unclear	Unclear	Low	Low	Low	Unclear
Sarkar et al. 2014 [18]	Unclear	Unclear	Low	Low	Low	Unclear
Shah et al. 2015 [19]	Low	Unclear	Low	Low	Low	Unclear
Sharma et al. 2016 [20]	Unclear	Low	Low	Low	Low	Low
Williams et al. 2012 [21]	Unclear	Low	Low	Low	Low	Unclear

## Supplementary Materials

Supplementary Materials Supplementary Table  1. Search strategy for PubMed and EMBASE.
