# Supplementary material for: Crystalloid Coload Reduced the Incidence of Hypotension in Spinal Anesthesia for Cesarean Delivery, When Compared to Crystalloid Preload: A Meta-Analysis
Source: Biomed Res Int. 2017 Dec 17;2017:3462529. doi: 10.1155/2017/3462529 (PMC5748285; doi:10.1155/2017/3462529)
Supplement: Supplementary Materials — Supplementary Table 1. Search strategy for PubMed and EMBASE. [file 3462529.f1.pdf]

Supplementary Table 1. Search strategy for PubMed and EMBASE

PubMed

Searched on: April 17, 2017

Results: 202

Search	Query
#1	"Cesarean Section"[mh]
#2	(Cesarean Sections[Title/Abstract]) OR Delivery, Abdominal[Title/Abstract]) OR Abdominal Deliveries[Title/Abstract]) OR Deliveries, Abdominal[Title/Abstract]) OR Caesarean Section[Title/Abstract]) OR Caesarean Sections[Title/Abstract]) OR Abdominal Delivery[Title/Abstract]) OR C-Section (OB)[Title/Abstract]) OR C Section (OB)[Title/Abstract]) OR C-Sections (OB)[Title/Abstract]) OR Postcesarean Section[Title/Abstract])
#3	#1 OR #2
#4	"Fluid Therapy"[mh]
#5	(Therapy, Fluid[Title/Abstract]) OR Fluid Therapies[Title/Abstract]) OR Therapies, Fluid[Title/Abstract]) OR Oral Rehydration Therapy[Title/Abstract]) OR Therapy, Oral Rehydration[Title/Abstract]) OR Rehydration Therapy, Oral[Title/Abstract]) OR Oral Rehydration Therapies[Title/Abstract]) OR Rehydration Therapies, Oral[Title/Abstract]) OR Therapies, Oral Rehydration[Title/Abstract]) OR Rehydration[Title/Abstract]) OR Rehydrations[Title/Abstract]) OR Oral Rehydration[Title/Abstract]) OR Oral Rehydrations[Title/Abstract]) OR Rehydrations, Oral[Title/Abstract]) OR Rehydration, Ora[Title/Abstract])
#6	#4 OR #5
#7	crystalloid
#8	(coload[Title/Abstract]) OR co-load[Title/Abstract]) OR coloading[Title/Abstract]) OR co-loading[Title/Abstract])
#9	(load[Title/Abstract]) OR loading[Title/Abstract]) OR preload[Title/Abstract]) OR preloading[Title/Abstract]) OR pre-loading[Title/Abstract])

#10	#6 OR #7 OR #8 OR #9
#11	(randomized controlled trial[Publication Type] OR randomized[Title/Abstract] OR placebo[Title/Abstract])
#13	#3 AND #10 AND #11

EMBASE

Searched on: May 4, 2017

Results: 52

**Search**	**Query**
#1	'cesarean section'/exp
#2	'caesarean sections':ab,ti
#3	'delivery, abdominal':ti,ab
#4	'abdominal deliveries':ti,ab
#5	'c-sections':ti,ab
#6	'c-section':ti,ab
#7	#1 OR #2 OR #3 OR #4 OR #5 OR #6
#8	'therapy, fluid':ti,ab
#9	'fluid therapies':ti,ab
#10	'therapies, fluid':ti,ab
#11	'therapy, oral rehydration':ti,ab
#12	'rehydration therapy, oral':ti,ab
#13	'oral rehydration therapies':ti,ab
#14	'rehydration therapies, oral':ti,ab
#15	'therapies, oral rehydration':ti,ab
#16	'rehydration':ti,ab
#17	'rehydration, oral':ti,ab
#18	'oral rehydrations':ti,ab
#19	'oral rehydration':ti,ab
#20	'rehydrations':ti,ab

#21	#8 OR #9 OR #10 OR #11 OR #12 OR #13 OR #14 OR #15 OR #16 OR #17 OR #18 OR #19 OR #20
#22	'load':ti,ab
#23	'loading':ti,ab
#24	'preload':ti,ab
#25	'preloading':ti,ab
#26	'pre-loading':ti,ab
#27	'coload':ti,ab
#28	'co-load':ti,ab
#29	'coloading':ti,ab
#30	'co-loading':ti,ab
#31	#22 OR #23 OR #24 OR #25 OR #26 OR #27 OR #28 OR #29 OR #30
#32	'crystalloid':ti,ab
#33	#21 OR #31 OR #32
#34	'random':ti,ab
#35	'placebo':ti,ab
#36	'double-blind':ti,ab
#37	#34 OR #35 OR #36
#38	#7 AND #33 AND #37
